# Unveiling the Therapeutic Potential and Healthcare Applications of Marine Therapy: A Systematic Review with Meta-Analysis and Meta-Regression

**DOI:** 10.3390/md21120604

**Published:** 2023-11-23

**Authors:** Sung Ryul Shim, Dayeon Shin, Seong-Jang Kim, Young Kook Kim, Kyung Ju Lee

**Affiliations:** 1 Department of Biomedical Informatics, College of Medicine, Konyang University, Daejeon 35365, Republic of Korea; sungryul.shim@gmail.com; 2 Department of Food and Nutrition, Inha University, Incheon 22212, Republic of Korea; dyshin@inha.ac.kr; 3 Department of Nuclear Medicine, College of Medicine, Pusan National University, Yangsan 50612, Republic of Korea; growthkim@pusan.ac.kr; 4 BioMedical Research Institute for Convergence of Biomedical Science and Technology, Pusan National University Yangsan Hospital, Yangsan 50612, Republic of Korea; 5 Department of Ophthalmology, College of Medicine, Seoul National University Hospital, Seoul 03080, Republic of Korea; eyedry@snu.ac.kr; 6 EyeLight Data Science Laboratory, Seoul 03080, Republic of Korea; 7 Department of Women’s Rehabilitation, National Rehabilitation Center, Seoul 01022, Republic of Korea

**Keywords:** marine therapy, marine-derived resources, seawater, mineral water, ocean therapy

## Abstract

This study was conducted to estimate the effectiveness of marine-derived resources for treating specific diseases, as well as identify the most effective methods for applying such resources in therapeutic applications. Bibliographic databases (PubMed, Embase, and Cochrane) were searched from their inception until May 2023 using Medical Subject Headings terms and text keywords related to seawater, mineral water, or ocean therapy. Fifteen eligible studies were included, involving 1325 participants aged 42.7–63.0 years. In the subgroup analysis based on treatment type, the mean difference was −1.581 (95% CI: −1.889, −1.274) for seawater with sun exposure and −1.210 (95% CI: −1.417, −1.002) for seawater with sun exposure, mud pack application, and sulfur pool therapy. The pooled standardized mean difference was calculated for different outcomes; the results were −1.110 (95% CI: −3.028, 0.806) for osteoarthritis severity, −0.795 (95% CI: −0.982, −0.607) for arthritis pain, −1.623 (95% CI: −2.036, −1.209) for fibromyalgia pain, and −1.498 (95% CI: −1.888, −1.108) for quality of life. Marine therapy is, therefore, promising for treating chronic skin issues, easing musculoskeletal discomfort, and enhancing the quality of life among patients with musculoskeletal pain.

## 1. Introduction

Marine therapy refers to the utilization of marine-derived resources for healthcare applications. It has received attention as a potential avenue to enhance holistic physical and mental wellness, including the utilization of natural elements such as saltwater, seaweed, mud, and other organic materials.

In this current age marked by aging populations, morbidity and mortality surged during the COVID-19 pandemic, bringing attention to individualized healthy lifestyle strategies within the framework of public health [1,2,3]. This approach seeks to alleviate mental and physical burdens, improve immunological function, and prevent chronic diseases and disabilities, ultimately leading to increased life expectancy.

Longevity or an extended lifespan leads to individuals encountering age-associated health challenges including chronic illnesses and disabilities. The World Health Organization has reported that noncommunicable diseases (NCDs)—including heart disease, stroke, cancer, diabetes, and chronic lung disease—are responsible for approximately 410 million deaths worldwide each year [4]. Certain vulnerable populations, such as the elderly and individuals with weakened immune systems, may be more susceptible to risk factors contributing to NCDs [4,5]. Fortunately, these NCDs are preventable and manageable.

A recent review of the link between the ocean and human health highlighted growing evidence of the ocean’s impact on human wellbeing [6,7]. Utilizing marine resources—including water, mud, and other natural substances—has the potential to contribute to healthy lifestyles and may offer therapeutic advantages. Ongoing research is exploring the potential therapeutic uses of these marine-derived resources in healthcare.

The requirement for a comprehensive literature summary on marine therapy encompassing seawater therapy with sun, sulfur pools, or mud is crucial, owing to their significance as keywords for conducting meta-analyses. Research on water-related marine therapy has consistently demonstrated its efficacy and contribution to health and wellbeing; this has been substantiated by various systematic reviews. For example, aquatic exercises have been shown to effectively enhance glycemic control in adults with type 2 diabetes [8]. Moreover, they have shown the potential to increase fitness levels and enhance the overall quality of life (QoL) in various adult populations [9]. A distinct advantage of water-based exercise is its ability to increase intensity while minimizing stress. Peloid therapy, a form of marine therapy involving mud immersion, has demonstrated favorable outcomes in chronic arthropathy and has been associated with lower arterial blood pressure values [10]. Balneotherapy, a bath therapy in which the head is immersed in warm mineral water, improves sleep quality and alleviates mental stress and chronic skin diseases [11]. Psoriasis is an immune-mediated chronic inflammatory skin disease that can also involve joints [12]. Patients who have undergone marine therapy have reported a significant improvement in their quality of life along with a marked improvement in their physical skin condition [13,14]. Additionally, climate therapy, safe treatment alternatives, variations in humidity and air quality, and fluctuations in barometric pressure have demonstrated positive effects on chronic skin conditions, contributing to an enhanced QoL [15].

The objective of this study was to explore the relationship between oceanic environmental factors—particularly water, sunlight, and mud—and human health, with a specific emphasis on their therapeutic applications. Our research questions were as follows: What is the effectiveness of utilizing marine-derived resources to treat specific diseases? Furthermore, which methods are preferred or most effective in such applications?

## 2. Materials and Methods

This study adhered to the Meta-analysis of Observational Studies in Epidemiology (MOOSE) reporting guidelines [16]. The study protocol related to this research was registered in PROSPERO (registration number CRD42023452677).

### 2.1. Data Sources and Literature Searches

A thorough search of the PubMed, Embase, and Cochrane databases was performed using Medical Subject Heading terms and text keywords from the start of the databases to May 2023 (Table S1). Subject headings and text keywords were related to seawater, mineral water, or ocean therapy. The search terms were categorized using Boolean operators (e.g., AND, OR, and NOT). This search was conducted without regard to language or study type. Two independent researchers (S.R.S. and K.J.L.) supplemented the search by manually examining trial databases and reference lists to identify additional relevant studies.

### 2.2. Study Selection

The study inclusion criteria were as follows: (1) studies including participants with or without a specific disease; (2) studies on interventions, including seawater therapy only as well as seawater therapy with sun, sulfur pool, or mud; (3) if the study did not show a difference in effect size, we analyzed the mean difference before and after treatment; and (4) studies including outcomes measured as mean differences in severity and pain of diseases. To ensure data accuracy and relevance, duplicate publications and articles without original data (e.g., case reports, abstracts only, review articles, editorials, and letters) were excluded. Furthermore, studies with fewer than two diseases or outcome groups were excluded from the analysis. The titles, abstracts, and full-text articles were independently evaluated by two investigators (S.R.S. and K.J.L.) according to the predetermined inclusion and exclusion criteria. Data extraction was performed by the authors using dedicated data extraction forms, and article inclusion was confirmed through a collaborative evaluation discussion involving all investigators. To ensure the accuracy and integrity of the meta-analysis, references and data from each included study were thoroughly examined to eliminate any potential overlaps.

### 2.3. Data Extraction

The basic details of the studies (first author, publication year, study design, treatments, diseases, outcome measures, and treatment duration) and patient characteristics (number of patients, age, and female sex) were extracted from the included articles using a predetermined data extraction form. In cases where the studies did not report standard deviations, a combined standard deviation was estimated for the two groups. The final meta-analysis included only studies that provided comprehensive information.

### 2.4. Statistical Analysis

To measure the effect of seawater therapy, Hedges’ g (or standardized mean differences (SMDs)), along with their 95% confidence intervals (CIs) for severity, pain, and QoL, were calculated for continuous variables. To adequately estimate the overall effect sizes, SMDs with their corresponding 95% CIs were calculated using fixed- or random-effects models, depending on the model assumptions [17]. More specifically, a random-effects model was used when the I^2^ statistic was >50%, and a fixed-effects model was used when the I^2^ statistic was <50%. A random-effects model analyzed using a restricted maximum-likelihood (REML) estimation was used to obtain the pooled overall SMDs and 95% CIs for outcomes [18].

A meta-regression analysis was performed for moderators comprising continuous variables, such as the number of patients, treatment duration, age, and proportion of females. Additionally, meta-ANOVA was conducted for categorical variables, including disease group, treatment type, and quality assessment group. The REML estimator was used to evaluate the variance of the true effects to analyze potential moderators. A two-sided *p*-value ≤ 0.05, or absence of a null value (SMD = 0) within the 95% CI, was considered significant. The analysis was conducted using the R software (version 4.3.1; R Foundation for Statistical Computing) [17].

### 2.5. Assessment of Potential Publication Bias

A funnel plot was created to assess the potential publication bias. The funnel plot utilized the standard error as a measure of the study size and plotted the SMDs before and after seawater therapy. In the absence of publication bias, studies typically demonstrate a symmetrical distribution based on the combined effect sizes. To further evaluate publication bias, we performed Egger’s linear regression test, as well as the Begg and Mazumdar rank correlation test [17].

### 2.6. Quality Assessment

The Newcastle–Ottawa quality scale was used to evaluate the quality of the observational studies [19]. We assessed the following three parameters: (1) appropriate selection; (2) comparability of the research design or statistical analysis; and (3) outcome/exposure ascertainment and research procedures. We graded each parameter with a star; a study can be awarded a maximum of one star per item for selection and outcome/exposure, and a maximum of two stars for comparability. The quality of the evidence related to the estimation of benefits and disadvantages was displayed according to the specific conditions [19].

## 3. Results

### 3.1. Study Selection

**Figure 1:**
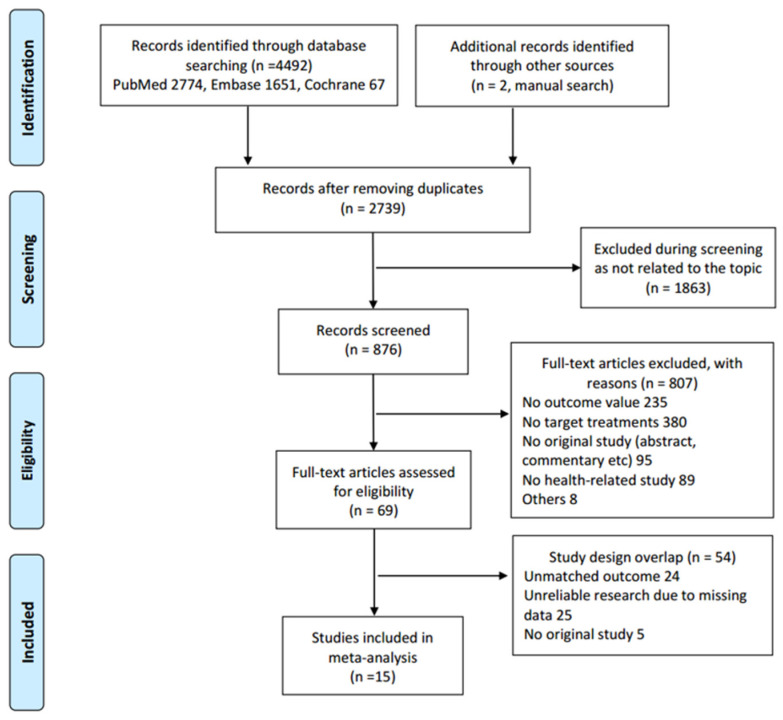
Preferred reporting items for systematic reviews and meta-analyses (PRISMA) flow diagram of studies included in the selection process.

The initial search yielded 4494 articles from PubMed (*n* = 2774), Cochrane (*n* = 67), and Embase (*n* = 1651). Of these, 1755 studies were excluded as they either contained overlapping data or appeared in multiple databases. Following title and abstract screening, 1863 studies were eliminated as they were not related to the topic or consisted solely of abstracts. Among the 69 full-text articles assessed, 54 were excluded owing to unmatched outcomes (*n* = 24), unreliable research (*n* = 25), or not being an original study (*n* = 5). Ultimately, 15 studies met the selection criteria for qualitative and quantitative analyses (Figure 1).

To examine the specific variations and participant descriptions outlined in Table 1, systematic reviews and meta-analyses were conducted on 15 studies [13,14,20,21,22,23,24,25,26,27,28,29,30,31,32] involving 1325 participants. All of the studies, except one by Harari et al. (2012) [26], were prospective observational studies, and all of the studies except one by de Andrade et al. (2008) [23] were conducted in the Dead Sea in Israel. Treatments included seawater only, seawater with sunlight, and seawater with sunlight and mud. Diseases included atopic dermatitis, psoriasis and psoriatic arthritis, psoriatic arthritis, fibromyalgia, and rheumatoid arthritis. The mean age ranged from 42.7 to 63.0 years, the proportion of females ranged from 4.5 to 88.9%, and the treatment period ranged from 2 to 12 weeks (Table 1).

### 3.2. Outcome Findings

**Figure 2:**
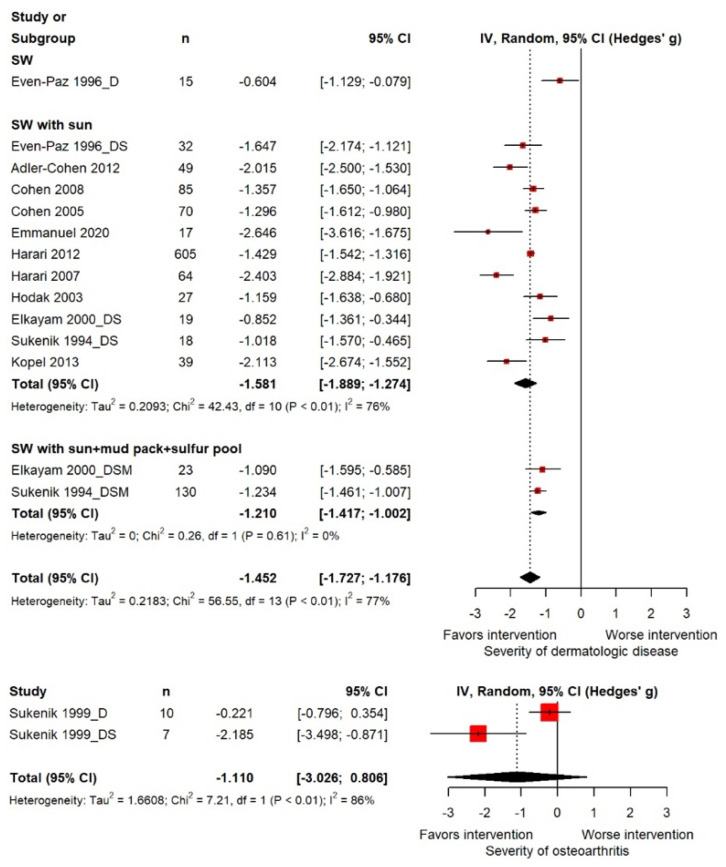
Forest plot of the standardized mean difference for the effect of seawater therapy on the severity of diseases.

The pooled SMD for the objective severity of dermatological diseases was −1.452 (95% CI: −1.727, −1.176), which was statistically significant. The heterogeneity test demonstrated significance at *p* < 0.01, and the Higgins I^2^ value was 77.0%. In the subgroup analysis by treatment type, the mean difference was −1.581 (95% CI: −1.889, −1.274) for seawater with sun and −1.210 (95% CI: −1.417, −1.002) for seawater with sun exposure, mud pack application, and sulfur pool therapy (Figure 2). The pooled SMD for the severity of osteoarthritis was −1.110 (95% CI: −3.028, 0.806), which was not statistically significant. The heterogeneity test demonstrated significance at *p* < 0.01, and the Higgins I^2^ value was 86.0% (Figure 2).

**Figure 3:**
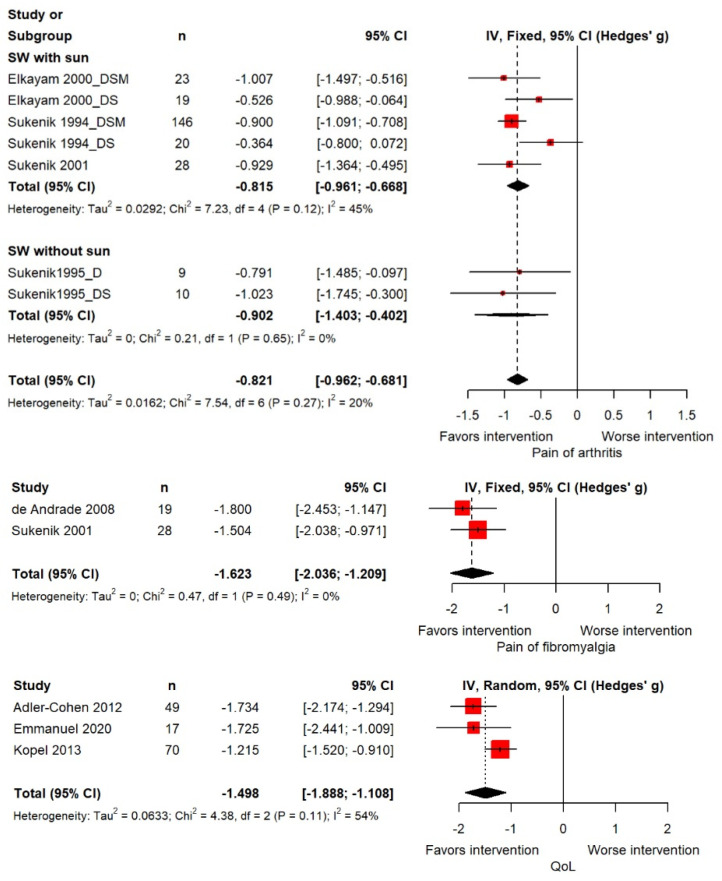
Forest plot of the standardized mean difference for the effect of seawater therapy on pain of diseases and quality of life.

The pooled SMD for arthritis pain was −0.821 (95% CI: −0.962 to −0.681), which was statistically significant. The heterogeneity test demonstrated significance at *p* = 0.27, and the Higgins I^2^ value was 20.0%. In the subgroup analysis by treatment type, the mean difference was −0.815 (95% CI: −0.961, −0.668) for seawater with sun treatment and −0.902 (95% CI: −1.403, −0.402) for seawater only (Figure 3). The pooled SMD for fibromyalgia pain was −1.623 (95% CI: −2.036, −1.209), which was statistically significant. The heterogeneity test demonstrated significance at *p* = 0.49, and the Higgins I^2^ value was 0.0%. The pooled SMD for QoL was −1.498 (95% CI: −1.888 to −1.108), which was statistically significant. The heterogeneity test demonstrated significance at *p* = 0.11, and the Higgins I^2^ value was 54.0% (Figure 3).

### 3.3. Moderator Analysis

This study explored the potential moderating roles of specific variables through meta-regression and meta-analysis of variance models (Table 2). Statistically significant differences were observed between the treatment groups (*p* = 0.005), with a significantly greater reduction in severity observed in the seawater with sun group −1.581 (95% CI: −1.889, −1.274). When the severity was analyzed by disease group, the *p*-value demonstrated borderline statistical significance. Atopic dermatitis (−2.015; 95% CI: −2.500, −1.530) showed the greatest improvement, followed by psoriasis (−1.513; 95% CI: −1.912, −1.113), and psoriasis and psoriatic arthritis (−1.252; 95% CI: −1.647, −0.856). No significant differences were observed among the remaining covariates for the number of patients, treatment duration, or quality assessment of the individual studies.

### 3.4. Publication Bias

The statistical methods employed to detect publication bias or small-study effects are shown in PubMed and Cochrane Library (Table S1). The SMDs for the objective severity of dermatological diseases were generally symmetrical, although four and two studies on the left and right side, respectively, were outside the funnel. The SMDs for arthritis pain were evenly distributed from side to side within the funnel plot, giving a visually symmetrical graph. Egger’s linear regression test (*p* = 0.699) and the Begg and Mazumdar rank correlation test (*p* = 0.869) suggested no evidence of publication bias or small-study effects in this meta-analysis.

### 3.5. Quality Assessment

We critically appraised the selected 15 studies using the criteria given by the Newcastle–Ottawa quality scale, and the final quality evaluation was discussed among all investigators (Embase (Table S1)). Five studies were ranked to be of good quality (Harari et al., 2012 [26], de Andrade et al., 2008 [23], Elkayam et al., 2000 [24], Sukenik et al., 1995 [32], and Sukenik et al., 1994 [31]), and five studies were ranked to be of fair quality (Kopel et al., 2013 [14], Adler-Cohen et al., 2012 [20], Harari et al., 2007 [27], Hodak et al., 2003 [28], and Sukenik et al., 2001 [29]). Five studies (Emmanuel et al., 2020 [13], Cohen et al., 2008 [21], Cohen et al., 2005 [22], Sukenik et al., 1999 [30], and Even-Paz et al., 1996 [25]) were ranked as poor quality owing to the relatively small sample size, lack of representativeness for the exposed cohort, and selection of the nonexposed cohort.

## 4. Discussion

This study aimed to determine the effectiveness of marine therapy for diseases and treatments, and we proposed the hypothesis that marine therapy interventions would effectively help in managing the objective severity of atopic dermatitis. Marine therapy is a therapeutic approach that uses oceanic and other aquatic resources to assist in the enhancement of individuals’ wellbeing. In this study, we explored marine-derived resources—including water, sunlight, and mud—as therapeutic agents for specific diseases, such as psoriasis, atopic dermatitis, and arthritis, as well as their preferred or most effective applications. Additionally, the results of this study provide information regarding possible target populations—such as middle-aged and elderly females with chronic diseases—and the feasible benefit of longer therapy time.

Psoriasis, atopic dermatitis, and arthritis are characterized by their chronic nature and high incidence rates [12,33,34]. The treatment options for these conditions vary widely, ranging from topical or localized treatments to systemic treatments involving small molecules and biological therapies [35]. Among the causes of these conditions, immune suppression can be noted, which necessitates extensive and prolonged use of steroids [36,37]. The resulting side effects contribute significantly to the increased occurrence of NCDs in conjunction with aging [38]. These conditions are highly influenced by the surrounding environment and lead to deterioration in QoL [39,40,41].

In this sense, as demonstrated in our review, marine therapy can reduce the severity of symptoms and pain and improve QoL. An advantage of this study is that it demonstrates the variations in effectiveness based on the combination of marine-derived resources. The methods employed to alleviate these conditions or symptoms were largely seawater with sun exposure, aquatic exercise in seawater, mud pack application, and sulfur pool therapy. It is likely that the effects of marine therapy on disease severity differ significantly depending on the method employed. Furthermore, the influence of the intervention is likely to vary more significantly, in the order of seawater with sun exposure (−1.581 [95% CI: −1.889, −1.274]), seawater with sun exposure and mud pack application (−1.210 [95% CI: −1.417, −1.002]), and seawater alone (−0.604 [95% CI: −1.129, −0.079]). The effectiveness of the intervention for pain demonstrated a statistically significant impact when marine therapy-related water was considered as an individual factor. Additionally, the interventions proved effective for conditions such as psoriatic arthritis and fibromyalgia, with the combination of seawater and sunlight proving effective during treatment. However, when the subgroups were examined, no significant covariates acted as moderators.

The marine therapy employed in this study was mostly conducted in the Dead Sea. With its long history, the Dead Sea is characterized by the presence of rich salts, mineral content, and consistent haze, which reduces potential exposure to harmful solar ultraviolet radiation. The Dead Sea is located at the Earth’s lowest inhabited point, situated 419 m below sea level, and its unique combination of features has led to its widespread use in disease management and treatment [25,29,42,43].

This study has several strengths; first, it is the first systematic review and meta-analysis to evaluate the relationships among marine therapy, disease severity, and QoL. Second, we considered the discrepancies among included studies, particularly those related to the severity and pain associated with the utilization of water and marine-derived resources. Still, this study had several limitations; one is that most of the research was conducted in the Dead Sea, which may introduce regional bias and restrict the scope to chronic skin diseases and musculoskeletal pain. Additionally, although the study was prospective, it only compared marine therapy using seawater with basic marine-derived resources.

In the future, it is essential to expand research efforts beyond the scope of the Dead Sea environment; this includes conducting randomized controlled trials in different settings to broaden our understanding. There is a significant need for research that delves into the various health conditions and symptoms, particularly in the context of public healthcare. Being primarily supplementary, marine therapy often demands prolonged application and the development of more effective approaches tailored to specific health needs, including appropriate therapy duration. The key lies in conducting well-organized systematic studies that carefully consider these factors to ensure the best outcomes for individuals seeking such therapies.

In conclusion, marine therapy shows promise as a viable therapeutic approach for improving chronic skin conditions and alleviating musculoskeletal discomfort. The outcomes of our meta-analysis and meta-regression highlight that the most effective intervention for atopic dermatitis involves combining seawater with sun exposure, which significantly improves the condition when marine-derived resources are utilized. Additionally, in the case of arthritis-related pain, the use of seawater with sun exposure has proven notably effective in reducing discomfort. Applying marine therapy to individuals with musculoskeletal pain has demonstrated substantial improvements in their QoL.

## Figures and Tables

**Table 1:** Characteristics of the studies included in the systematic review.

Study	Study Design	Mean Age (Years)	Female Rate	No. of Participants	Treatments (n)	Disease	Outcome Measures	Treatment Duration (Weeks)
Emmanuel et al. [17]	Prospective cohort study	52.2	35.3%	17	Seawater with sun (17)	Psoriasis	Severity (PASI), QoL (DLQI)	4
Kopel et al. [18]	Prospective study	46.8	48.6%	70	Seawater with sun	Psoriasis and psoriatic arthritis	Severity (PASI), QoL (Skindex-29)	4
Adler-Cohen et al. [19]	Prospective study	40.6	49.0%	49	Seawater with sun (49)	Atopic dermatitis	Severity (SCORAD), QoL (Skindex-29)	4
Harari et al. [20]	Retrospective study	48.1	4.5%	605	Seawater with sun	Psoriasis	Severity (PASI)	4
Cohen et al. [21]	Prospective study	52.5	44.7%	85	Seawater with sun	Psoriasis	Severity (PASI)	2
de Andrade et al. [22]	Prospective study	48.5	50.0%	19	Aquatic exercise in the seawater	Fibromyalgia	Pain (no. of active joints)	12
Harari et al. [23]	Prospective study	42	34.4%	64	Seawater with sun	Psoriasis	Severity (PASI)	4
Cohen et al. [24]	Prospective study	48.5	42.9%	70	Seawater with sun	Psoriasis	Severity (PASI)	2
Hodak et al. [25]	Prospective study	48.5	33.3%	27	Seawater with sun	Psoriasis	Severity (PASI)	4
Sukenik et al. [26]	Prospective study	48.5	33.9%	56	Seawater with sun (28), seawater with sun, mud pack, and sulfur pool (28)	Psoriatic arthritis and fibromyalgia	Pain (no. of active joints)	3
Elkayam et al. [27]	Prospective study	52	38.1%	42	Seawater with sun (19), seawater with sun, mud pack, and sulfur pool (23)	Psoriasis and psoriatic arthritis	Severity (PASI), pain (no. of active joints)	4
Sukenik et al. [28]	Prospective study	63	88.9%	17	Seawater (10), seawater with sulfur pool (7)	Osteoarthritis_Kness	Severity (Lequesne index)	2
Even-Paz et al. [29]	Prospective study	48	50.0%	47	Seawater (15), seawater with sun (32)	Psoriasis	Severity (PASI)	4
Sukenik et al. [30]	Prospective study	60.1	86.1%	9	Seawater (9), seawater with sulfur pool (10)	Rheumatoid arthritis	Pain (no. of active joints)	2
Sukenik et al. [31]	Prospective study	42.7	50.7%	148	Seawater with sun (18), seawater with sun, mud pack, and sulfur pool (130)	Psoriasis and psoriatic arthritis	Severity (PASI), pain (no. of active joints)	3

PASI, Psoriasis Area and Severity Index; DLQI, Dermatology Quality of Life Index; SCORAD, Scoring Atopic Dermatitis index. The age in Cohen et al., 2005 [22], Hodak et al., 2003 [28], and Even-Paz et al., 1996 [25] was the median.

**Table 2:** Effects of moderators on severity and pain of diseases.

Severity							Pain					
Variables	*k*	Coef	SMD	95% CI		*p*	*k*	Coef	SMD	95% CI		*p*
No. of total participants	14	0.000		−0.002	0.002	0.979		−0.001		−0.005	0.002	0.451
Treatment duration	14	−0.154		−0.529	0.220	0.418		0.064		−0.261	0.389	0.699
Age	14	0.041		−0.028	0.109	0.249		−0.009		−0.044	0.026	0.610
Female rate	14	0.362		−1.935	2.659	0.757		−0.127		−1.392	1.138	0.844
Diseases						0.056						0.815
Psoriasis	8		−1.513	−1.912	−1.113							
Atopic dermatitis	1		−2.015	−2.500	−1.530							
Psoriasis and psoriatic arthritis	5		−1.252	−1.647	−0.856		4		−0.8	−0.955	−0.644	
Psoriatic arthritis and fibromyalgia							1		−0.929	−1.364	−0.495	
Rheumatoid arthritis							2		−0.902	−1.403	−0.402	
Treatments						0.005						0.635
Seawater	1		−0.604	−1.129	−0.079							
Seawater with sun	11		−1.581	−1.889	−1.274		5		−0.815	−0.961	−0.668	
Seawater with sun and mud	2		−1.210	−1.417	−1.002		2		−0.902	−1.403	−0.402	
Quality assessment						0.063						0.607
Poor and fair	9		−1.649	−2.050	−1.248		1		−0.929	−1.364	−0.495	
Good	5		−1.218	−1.430	−1.005		6		−0.809	−0.957	−0.66	

## Data Availability

Data supporting the findings of this study are available from the corresponding author upon reasonable request.

## Supplementary Materials

The following supporting information can be downloaded at: https://www.mdpi.com/article/10.3390/md21120604/s1; Table S1: Search queries.
