# Supplementary material for: Unveiling the Therapeutic Potential and Healthcare Applications of Marine Therapy: A Systematic Review with Meta-Analysis and Meta-Regression
Source: Mar Drugs. 2023 Nov 23;21(12):604. doi: 10.3390/md21120604 (PMC10744920; doi:10.3390/md21120604)
Supplement: Supplementary file 1 [file marinedrugs-21-00604-s001.zip › marinedrugs-2607565-supplementary.pdf]

Table S1. Search queries

PubMed and Cochrane Library

1. Therapeutic area; Ocean

("Oceans and Seas"[Mesh] OR "Seawater"[Mesh] OR "Seawater"[tiab] OR "Mineral Waters"[Mesh] OR "Mineral Waters"[tiab] OR "Dead sea"[tiab])

2. Intervention

("Therapeutics"[Mesh] OR "Balneology"[Mesh] OR "Balneology"[tiab] OR "Mud Therapy"[Mesh] OR "Mud Therapy"[tiab] OR "Climatotherapy"[Mesh] OR "Climatotherapy"[tiab] OR "Heliotherapy"[Mesh] OR "Heliotherapy"[tiab])

Embase

1. Therapeutic area; Ocean

('sea'/exp OR 'sea water'/exp OR 'mineral water'/exp OR sea:ab,ti OR ocean:ab,ti OR seawater:ab,ti OR 'mineral water':ab,ti)

2. Intervention

('therapy'/exp OR therapy:ab,ti OR treatment:ab,ti OR therapeutics:ab,ti OR 'sea therapy':ab,ti OR 'ocean therapy':ab,ti OR 'marine therapy':ab,ti OR 'climatotherapy'/exp OR 'thalassotherapy'/exp OR 'balneotherapy'/exp OR thalassotherapy:ab,ti OR balneotherapy:ab,ti OR crenobalneotherapy:ab,ti)
